# Correlations between negative life events and suicidal ideation among Chinese adolescents: a meta-analysis

**DOI:** 10.3389/fpsyt.2023.1201786

**Published:** 2023-09-14

**Authors:** Xubin He, Ping Yang, Qinyao Yu, Bo Yang

**Affiliations:** ^1^Chongqing Mental Health Center, Chongqing, China; ^2^Chongqing Medical School, Chongqing, China

**Keywords:** suicidal ideation, negative life events, adolescent, China, meta-analysis

## Abstract

**Background:**

Suicide ideation (SI) has become a serious social issue worldwide, and research has found a certain correlation between negative life events (NLE) and SI. Nevertheless, this relationship is still not clear among Chinese adolescents, a special population. Hence, this investigation performed a meta-analysis of observational research on the correlation between NLE and SI among adolescents in China, to further clarify the association.

**Methods:**

We performed an extensive search on seven electronic databases starting from their establishment until March 10, 2023. The research mainly focused on cross-sectional studies conducted on samples of Chinese adolescents. To examine the association between NLE and SI, a meta-analysis model using random effects was utilized. To investigate moderating factors such as age, region, assessment tools for SI, and year of publication, subgroup and meta-regression analyses were performed. The AHRQ evaluated the quality of the study. The synthesis of data was conducted utilizing STATA software (version 16).

**Results:**

Ultimately, a total of 30 cross-sectional studies were selected for this analysis, including 39,602 individuals in the participant sample. The results showed that NLE was moderately positively correlated with SI among Chinese adolescents (*r* = 0.29, 95% CI: 0.26, 0.32). In addition, this relationship was moderated by regional differences and the measurement tool used for SI. Studies conducted in Western China showed a higher correlation coefficient than those conducted in Eastern and Central China. Moreover, research conducted with the SSIOSS demonstrated a stronger correlation coefficient compared to studies utilizing the BSI-CV or other assessment instruments.

**Conclusion:**

This meta-analysis indicates that NLE is linked to SI in Chinese teenagers, especially those residing in Western regions of China. Identifying and intervening in NLE and associated risk factors are crucial to prevent suicide within this demographic.

## 1. Introduction

Globally, 703,000 people die by suicide every year, with a suicide-related death occurring in more than 1 out of every 100 deaths, and 77% of these suicides occur in low- and middle-income countries. Suicide has become the fourth leading cause of death for individuals aged 15–29 (1). Adolescence refers to the period between childhood and adulthood in the human lifespan, during which individuals typically experience the phase of sexual maturation (2). This period begins with the physical, cognitive, and social changes that occur at the onset of puberty. Emerging adults who transition out of adolescence must possess the skills necessary to navigate a complex society (3). Concurrently, adolescence is a period of rapid physical and psychological development (4). It is a stage characterized by significant development in terms of the body, brain, cognition, emotions, and behavior (5). However, this development can be strongly influenced by factors such as individual characteristics, family dynamics, community environments, and school contexts (6). When faced with academic, emotional, and interpersonal problems, adolescents may develop suicidal thoughts and behavior due to their psychological fragility and a lack of effective coping strategies and social support (7). Research indicates that approximately 67,000 adolescents worldwide die by suicide each year, with suicide being a leading cause of death among adolescents in middle- and low-income countries (8). Similarly, in China, which has the world’s largest population, suicide remains an important cause of death among adolescents (9) and is on the rise (10). Additionally, adolescent suicide often appears in clusters, with these individuals mainly concentrated in schools, making group institutions such as schools an important site for adolescent suicide (11). Therefore, adolescent suicide in Chinese schools has become an important public health research issue.

The World Health Organization defines suicidal behavior as a series of actions including suicidal ideation (SI), suicide planning, suicide attempt, and suicide death (12). SI is a fatal and inevitable stage of suicidal behavior and one of the most important warning indicators leading to subsequent suicide death (13, 14). SI refers to thoughts of taking action to end one’s life, including determining a method, formulating a plan, or having the intention to act (15). A study of 59 low- and middle-income countries found that the prevalence of SI among adolescents was 16.9% (16). Furthermore, research based on the global school-based student health survey showed that the past-year prevalence of SI among adolescents was as high as 17.7% (17). Similarly, 18%–25% of Chinese adolescents reported having experienced SI (18). Therefore, it is of great significance to explore the prevention of SI for suicide death and for SI itself.

The onset of Adolescent SI is impacted by several environmental, psychological, and biological variables (19). These factors can be categorized as potential or risk factors, which interplay with each other. However, due to intervention feasibility, researchers have increasingly focused on controllable risk factors, such as negative life events (NLE) within the living environment. NLE refers to events that occur in an individual’s daily life and harm the individual (20). These NLE are a predominant environmental risk factor, from adolescence through to adulthood. Studies indicate that NLE, such as academic pressure, failed romantic relationships, interpersonal conflicts, familial discord, and financial struggles, are significant drivers of psychological distress (21, 22). As adolescents spend a substantial portion of their time in social and educational domains, NLE in these areas is more common (23). NLE has been identified as a significant risk factor for SI (24). The research conducted by Musetti et al. (25) revealed a positive association between NLE and SI. Additionally, this relationship is influenced by attachment anxiety and reflective functioning. To date, four systematic reviews have been conducted to explore the association between NLE and SI. Of these, two were narrative reviews (26, 27). While they provided evidence of a correlation, the nature and strength of this association remain unclear due to limitations in the review methods employed. Franklin et al. (28) attempted a meta-analysis of the association’s strength but did not include a comprehensive analysis of adolescents as a distinct group. Additionally, a prospective systematic review and meta-analysis found that NLE increases the subsequent risk of SI. However, the number of included studies was limited, and there was a risk of publication bias, necessitating further research to enhance the reliability of these results (29). Moreover, previous reviews did not delve deeply into the specific population of adolescents. In the cultural context of China, prior studies have demonstrated a positive correlation between NLE and SI among Chinese adolescents (30). Nonetheless, Massing-Schaffer et al. (31) found no relationship between NLE and SI after controlling for depression. Furthermore, the strength of the relationship between NLE and SI varies significantly among Chinese adolescents, with correlation coefficients ranging from (*r* = 0.21) (30) to (*r* = 0.40) (32). So far, there has not been any meta-analysis conducted on the association between NLE and SI among adolescents in China. The objective of this study is to conduct a comprehensive analysis of previous studies examining the correlation between NLE and SI in Chinese teenagers. Moreover, the objective of this research is to investigate possible discrepancies in the correlation between NLE and SI among Chinese teenagers, considering factors like assessment methods, year of publication, and characteristics of the sample (age and geographic location). These variables possess the capability to impact the results of the meta-analysis (29). The study findings will provide insights for the development of targeted intervention programs in the future.

## 2. Methods

### 2.1. Materials and methods

This study adheres to the reporting guidelines outlined in the PRISMA framework (33). The research procedure has been properly recorded with PROSPERO and given the distinct registration code CD42023404819.

### 2.2. Search strategy

To examine the correlation between NLE and SI, seven databases underwent scrutiny. The Chinese databases consisted of China National Knowledge Infrastructure, Chinese Biomedical Literature Service System, Wanfang Database, and China Science and Technology Journal Database, whereas the English databases consisted of PubMed, Embase, and Web of Science. Research conducted until March 10, 2023, was explored in databases using both the Chinese and English languages. To search, we employed a mix of Medical Subject Headings (MeSH terms) and free-text terms, which were categorized into three main term groups (“life events,” “suicide,” and “adolescent”) (Supplementary material 1). In addition, to guarantee the inclusiveness of the literature, a manual examination was performed on the bibliographies of eligible studies and previous systematic reviews to discover possible studies for incorporation. The search encompassed articles published in both Chinese and English languages to encompass a broad spectrum of relevant research.

### 2.3. Study selection criteria

The inclusion criteria consist of the following: (1) reports on the association between NLE and SI, using Pearson or Spearman correlation coefficients (*r*); (2) samples consisting of Chinese adolescents aged 10–24 years (34); (3) literature types including cross-sectional, case-control, and cohort studies; (4) literature published in either Chinese or English; (5) no limitations on the particular scale employed to evaluate SI; (6) evaluation of NLE using the adolescent self-rating life events check list (ASLEC) developed by Liu et al. (35).

Exclusion criteria: (1) literature lacking adequate data for extraction; (2) literature categories encompassing literature reviews, conference abstracts, comments, and case reports; (3) duplicate publications, with the sample size determined by the largest study.

### 2.4. Data extraction

In this study, the NoteExpress software was utilized for the management and elimination of duplicate records in the collected literature. The screening and quality assessment of the articles were independently conducted by two trained researchers (XBH and QYY) using predefined inclusion and exclusion criteria. The entire process encompassed three stages. During the first stage, the titles and abstracts of all studies were independently screened to identify potentially eligible articles. Any discrepancies that arose were discussed, and consensus was reached through negotiations. In the second stage, further screening of the retained full-text articles was conducted. In cases of uncertainty, consultation with a third researcher (BY) was sought until a consensus was achieved. In the third stage, data extraction was primarily performed by (QYY), with comprehensive verification conducted by (XBH) to ensure accuracy. The data were cross-checked and allocated to a third investigator (BY). Simultaneously, in cases where acquiring the necessary data from the included literature posed challenges, the respective authors were contacted via email to obtain the required research data. The extracted data included author information, publication year, region, sample size, age, correlation coefficients (*r*-values), and measurement tools.

### 2.5. Quality assessment

To evaluate the standard of the cross-sectional studies incorporated in this analysis, we employed the criteria specified by the agency for healthcare research and quality (AHRQ). The quality of the study source, variables, timeframe, sample, bias, statistical analysis, data collection, and follow-up are assessed using a set of 11 criteria. The adequacy of the specific aspect was indicated by classifying each item as either “yes,” “no,” or “unclear.” A score of 1 point is given for “yes” answers, while “no” and “unclear” answers receive 0 points. The score varies between 0 and 11, with scores below 4 being classified as low quality, scores between 4 and 7 as moderate quality, and scores between 8 and 11 as high quality (36). Both researchers (XBH and QYY) autonomously conducted every phase of the quality evaluation procedure.

### 2.6. Statistical analysis

The statistical analysis was conducted utilizing STATA software (version 16.0). From each article, we acquired the correlation coefficient (*r*) using either Pearson or Spearman method. Afterward, we converted it into Fisher’s *Z* along with the standard error (SE). Next, the collective *r*-value was computed as the aggregated effect size, accompanied by a 95% confidence interval (95% CI). Finally, the pooled *r*-values and 95% CI were inverted to report the results for *r*. Heterogeneity was indicated if *p* < 0.05 or *I*^2^ > 50% (37). Subgroup analyses were conducted on various dimensions such as region, age, SI measurement tool, and year of publication to identify potential diverse factors that could impact the association between NLE and SI. To further investigate the origins of heterogeneity, meta-regression analysis, and evaluation tools are used to compare differences among subgroups. In this study, we followed the recommendation of Gignac and Szodorai (38) to consider *r* = 0.1, *r* = 0.2, and *r* = 0.3 as indicators of weak, moderate, and strong correlations, respectively. Moreover, a sensitivity analysis was performed to assess the impact of individual studies on the overall combined estimates. Funnel plots (39) and Egger’s statistics (40) were utilized for evaluating publication bias. If Egger’s test indicated statistical significance, a nonparametric pruning and padding analysis (41) was conducted. A significance level of less than 0.05 was deemed statistically significant.

## 3. Results

### 3.1. Characteristics and quality assessment of the studies included

**Figure 1:**
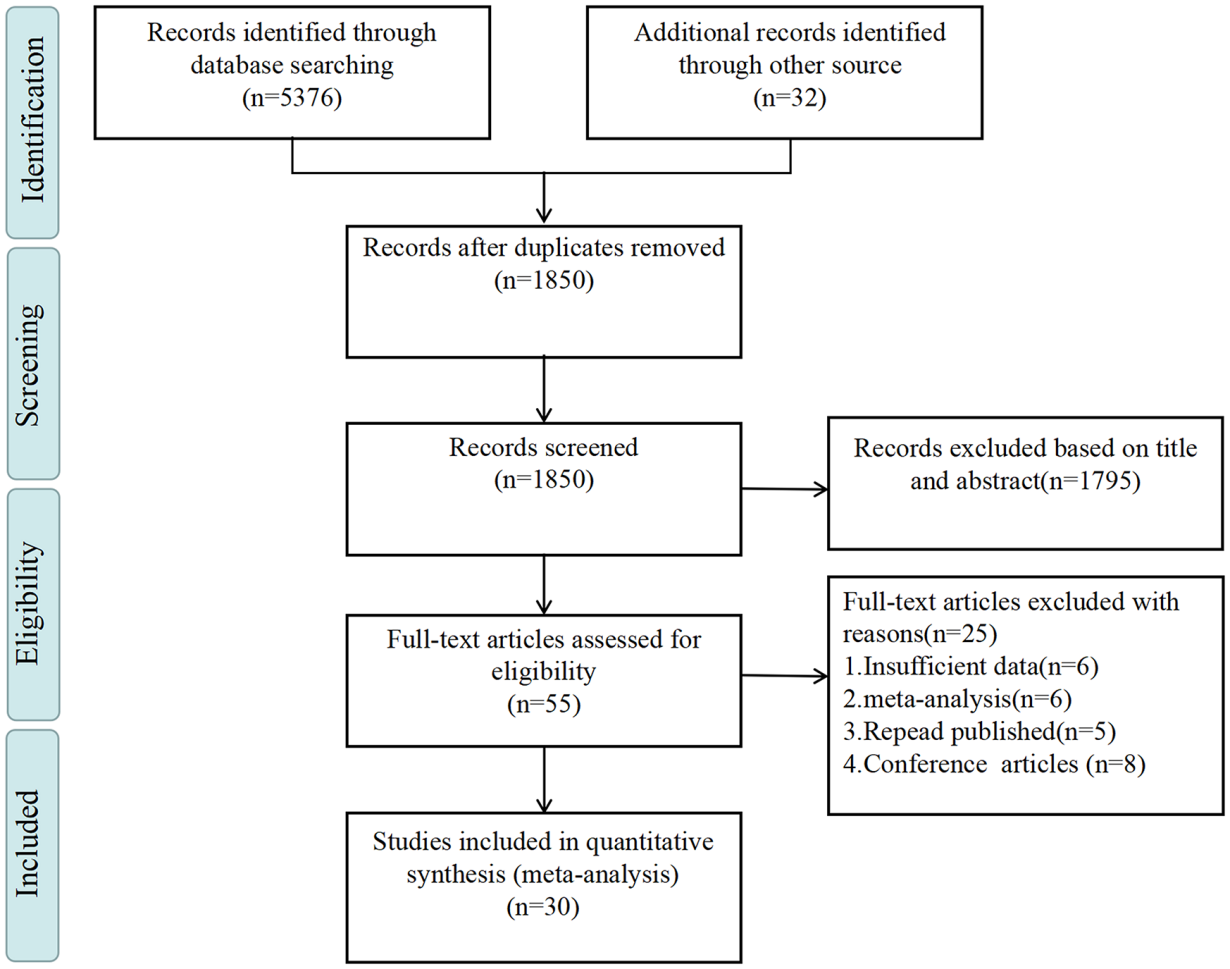
Literature selection process.

Based on the predetermined search plan, a total of 5,408 articles were discovered. After the initial review using NoteExpress, 1,850 papers were obtained. Following a comprehensive examination of the headings and summaries, a total of 55 articles were considered pertinent and chosen for complete textual perusal. Afterward, a total of 30 articles (30, 32, 42–69) were ultimately incorporated into the analysis.39,062 individuals were included in the study. Regarding the evaluation measures for SI, 12 studies employed the self-rating idea of the suicide scale (SSIOSS), which was created by Xia et al. (70), while 5 studies utilized the Beck scale for SI-Chinese version (BSI-CV). The remaining 13 studies employed various other instruments to measure SI. In order to minimize heterogeneity in the results caused by the assessment tools of NLE. Liu et al. (35) developed the ASLEC, which was used to evaluate the NLE. This scale comprises 27 items that capture negative life events capable of eliciting psychological reactions in adolescents. A rating on a scale of 0–5 is assigned to each item, indicating the event’s occurrence and impact on the adolescent in the previous year. The scale encompasses six factors: interpersonal relationships, academic stress, punishment, loss, and health adaptation. Among adolescents, it functions as a means of evaluating the occurrence and severity of life events associated with stress. Crucially, this measurement considers the distinct physiological and psychological traits of teenagers and their positions in both the family and society, rendering it highly suitable for Chinese youths in general. The size of the study populations in the articles included varied, with participant numbers ranging from 253 to 8,379. Quality assessment was conducted on all the literature included, with 7 studies being classified as high quality and 23 as moderate quality (Supplementary material 2). Table 1 presents literature characteristics, and Figure 1 illustrates the inclusion process.

**Table 1:** Characteristics of the included article.

References	Region	Sample size (male/female)	Age range/population	*r*	Type of assessment (SI)	Type of assessment (life events)	Study quality scores
Xiong and Deng (42)	Central	2,679 (1,183/1,496)	21.4 ± 1.6/C	0.12	BSI-CV	ASLEC	6
Duan (43)	Western	793 (358/435)	C	0.29	SIOSS	ASLEC	5
Chen (44)	Central	492 (157/199)	C	0.22	SIOSS	ASLEC	7
Yao (45)	Western	672 (279/393)	C	0.4	SSIOSS	ASLEC	6
Ma et al. (46)	Eastern	8,379 (4,016/4,363)	18.83 ± 1.69/C	0.29	BSI-CV	ASLEC	8
Xin and He (47)	Central	800 (390/410)	20.56 ± 1.58/C	0.31	SSIOSS	ASLEC	7
Chen et al. (48)	Central	732 (361/371)	20.42 ± 1.05/C	0.13	Other	ASLEC	5
Jiao et al. (49)	Central	504 (135/369)	18.78 ± 0.988/C	0.25	Other	ASLEC	8
Liang and Li (50)	Eastern	373 (180/193)	20.08 ± 1.46/C	0.3	Other	ASLEC	5
Zhou (51)	Eastern	408 (107/301)	C	0.14	Other	ASLEC	7
Xue and Liang (52)	Central	928 (348/580)	18.68 ± 3.42/C	0.39	SIOSS	ASLEC	5
Zhang and Chen (53)	Central	348 (166/182)	C	0.33	Other	ASLEC	5
Yang and Zhu (54)	Eastern	2,326 (769/1,557)	C	0.29	Other	ASLEC	6
Yang et al. (55)	Western	297 (218/81)	C	0.45	SIOSS	ASLEC	5
Zhang (56)	Eastern	1,034 (533/501)	M	0.32	Other	ASLEC	9
Li (57)	Central	866 (412/454)	17.2 ± 1.9/M	0.30	SIOSS	ASLEC	5
Wu et al. (58)	Central	2,471 (1,445/1,026)	19.7 ± 1.0/C	0.23	Other	ASLEC	9
Liu (59)	Eastern	253 (65/188)	C	0.18	BSI-CV	ASLEC	5
Fan and He (60)	Central	547 (153/394)	C	0.44	Other	ASLEC	7
Rong (61)	Central	1,197 (537/641)	18.58 ± 0.88/C	0.26	Other	ASLEC	5
Yang (62)	Eastern	670 (443/227)	19.66 ± 1.43/C	0.4	SSIOSS	ASLEC	6
Liu (63)	Eastern	1,171 (130/658)	C	0.31	BSI-CV	ASLEC	8
Chen et al. (64)	Eastern	2,802 (1,152/1,650)	C	0.18	Other	ASLEC	8
Xu (65)	Western	621 (245/376)	M	0.31	Other	ASLEC	5
Jiang et al. (66)	Eastern	499 (126/373)	C	0.32	SSIOSS	ASLEC	6
Ma et al. (67)	Western	1,468 (68/1,400)	19.81 ± 1.62/M	0.43	SSIOSS	ASLEC	7
Yang et al. (68)	Central	560	19.3 ± 1.3/C	0.24	SSIOSS	ASLEC	6
Wang et al. (32)	Western	894 (472/422)	18.88 ± 1.01/C	0.40	SSIOSS	ASLEC	7
Yang et al. (69)	Eastern	521 (115/406)	18.5 ± 0.8/C	0.28	BSI-CV	ASLEC	5
Yao et al. (30)	Eastern	5,211 (2,340/2,871)	20.74 ± 2.99/C	0.21	Other	ASLEC	8

C, college students; M, middle school student; SSIOSS, self-rating idea of suicide scale; BSI-CV, Beck scale for SI-Chinese version; other, other SI scale; ASLEC, adolescent self-rating life events check list.

### 3.2. Homogeneity test and meta-analysis

**Figure 2:**
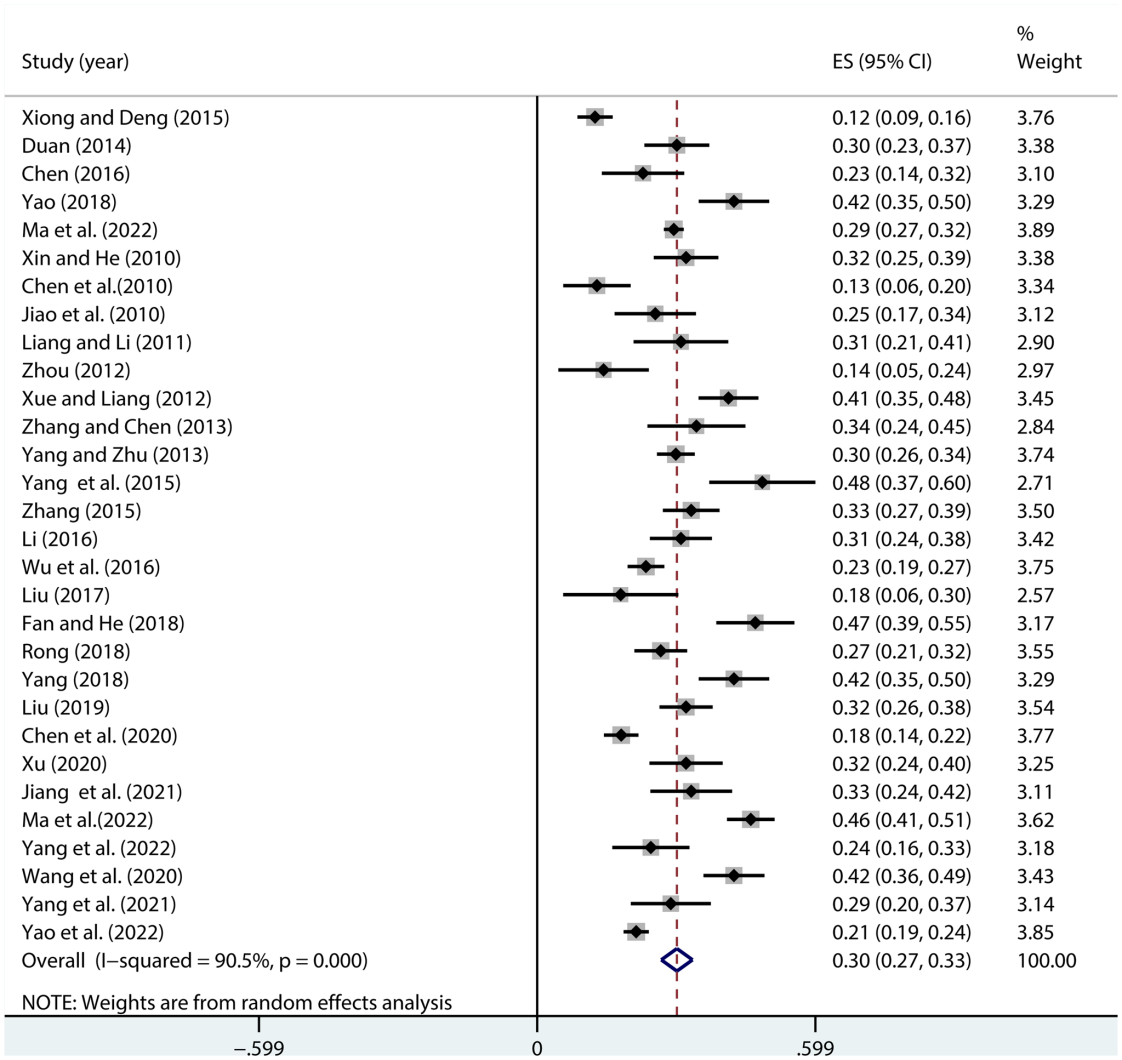
Forest plot of the relationship between NLE and SI.

Heterogeneity was found among the 30 studies that examined the association between NLE and SI (*I*^2^ = 90.5%, *p* < 0.001). The overall effect size for the random-effects model was *r* = 0.30 (95% CI, 0.27, 0.33), and *r* = 0.29 (95% CI, 0.26, 0.32) after transformation. A statistically significant correlation existed between NLE and SI (*Z* = 6.65; *p* < 0.001) (Figure 2).

### 3.3. Processing of the heterogeneity between IA and SI

The random-effects model indicated a notable amount of variability, prompting us to conduct subgroup and meta-regression analyses to examine and resolve this variability.

### 3.4. Subgroup analysis

Subgroup analysis was performed on the variables between NLE and SI: regional differences, age, year of publication, and SI measurement tools (Table 2).

**Table 2:** Subgroup analysis showing *r* of SI for NLE.

Variables	Included studies	95% CI for *r*		Heterogeneity test		Significance test	Meta-regression
		*r*	95% CI	*I*^2^ (%)	*p* ^a^	*Z*	*p* ^b^
**Region**							
Central	12	0.27	0.22, 0.33	90.4	0.010	9.04^**^	Ref
Eastern	12	0.27	0.24, 0.31	86.2	0.036	13.77^**^	0.942
Western	6	0.38	0.33, 0.43	74.8	0.004	13.33^**^	0.009
**Age**							
Middle school student	4	0.35	0.27, 0.41	83.3	<0.001	9.03^**^	Ref
College student	26	0.28	0.25, 0.32	89.8	<0.001	16.46^**^	0.221
**Publication year**							
2010–2016	15	0.27	0.22, 0.32	88.7	0.008	10.96^**^	Ref
2017–2023	15	0.31	0.27, 0.35	91.8	0.008	13.35^**^	0.231
**SI measures**							
SSIOSS	12	0.35	0.31, 0.39	78.8	0.005	15.53^**^	Ref
BSI-CV	5	0.24	0.16, 0.32	93.9	0.009	5.40^**^	0.015
Others	13	0.26	0.22, 0.30	85.4	0.004	12.68^**^	0.009

Ref, reference group; SSIOSS, self-rating idea of suicide scale; BSI-CV, Beck scale for SI-Chinese version; other, other SI scale.

a *p*-value for the heterogeneity within subgroups.

b *p*-value for the between-subgroup difference using meta-regression analysis; ^**^p < 0.001.

**Figure 3:**
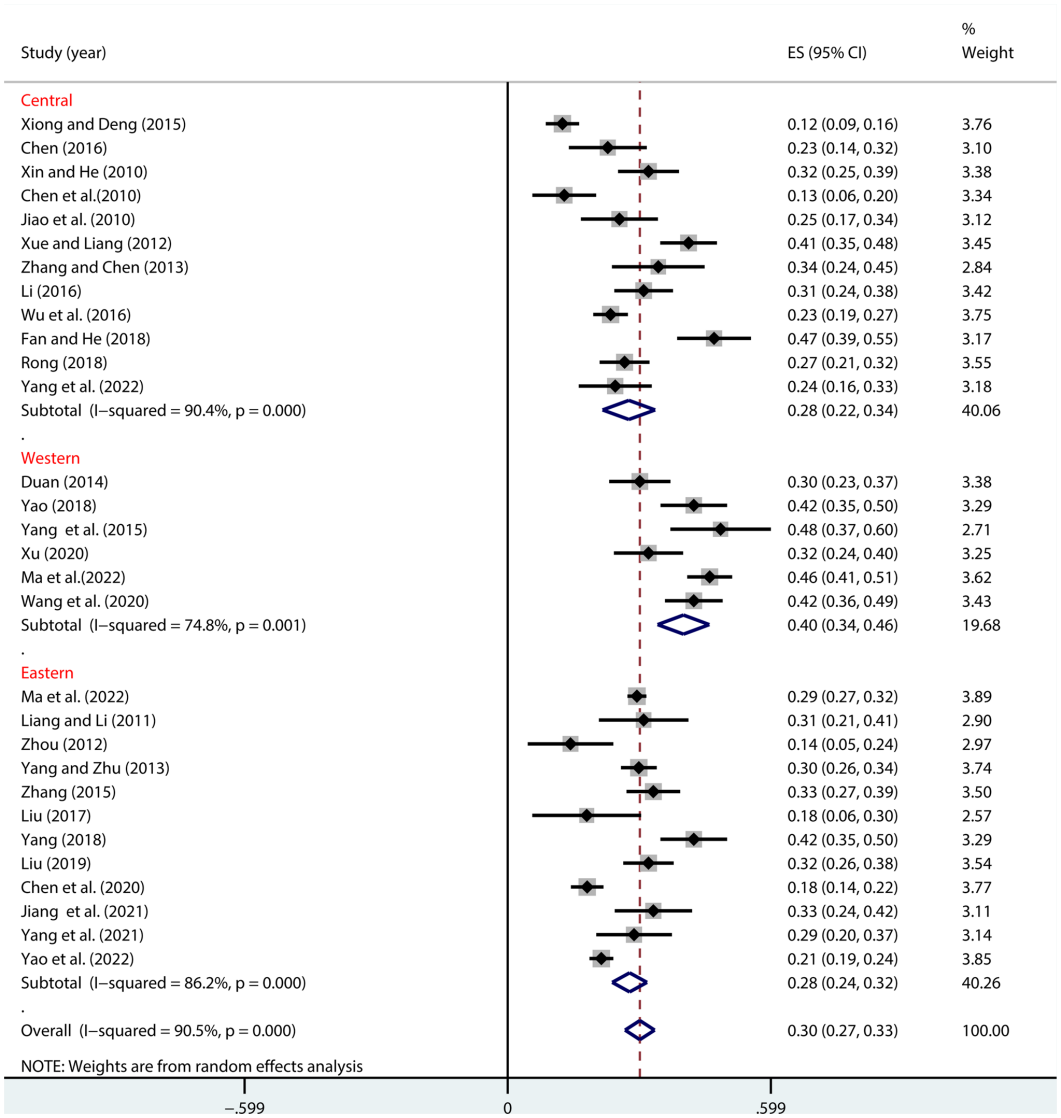
Moderation effect of regional differences on NLE and SI.

As shown in Figure 3, regional differences significantly moderated the relationship between NLE and SI (Table 2). The correlation was strongest in the Western region, with a transformed value of (*r* = 0.38, 95% CI: 0.33, 0.43). It was relatively smaller in the Eastern (*r* = 0.27, 95% CI: 0.24, 0.31) and Central regions (*r* = 0.27, 95% CI: 0.22, 0.33) (Figure 3).

**Figure 4:**
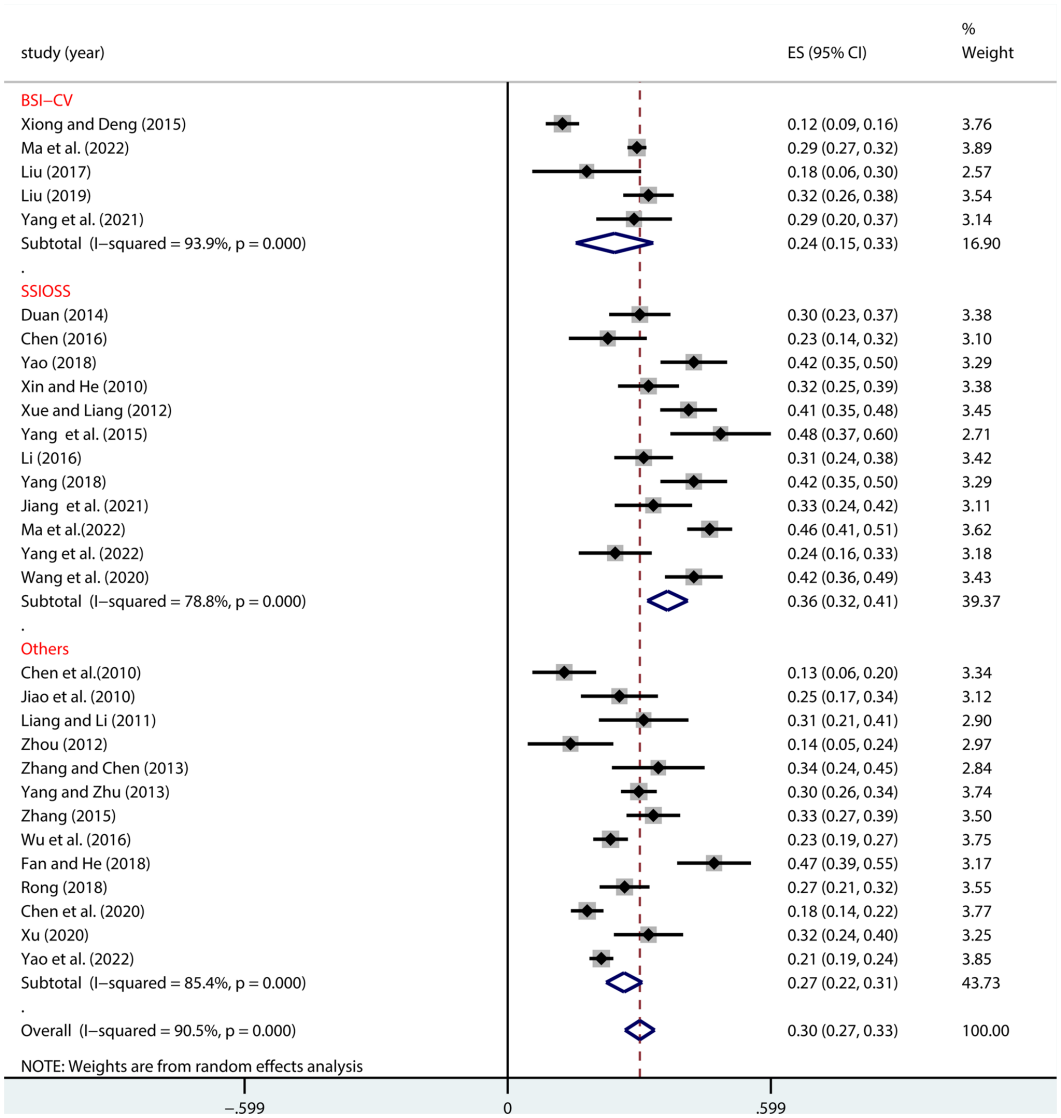
Moderation effect of SI assessment tool on NLE and SI.

As shown in Figure 4, the relationship between NLE and SI in Chinese adolescents was significantly moderated by the assessment tool (Table 2). This positive correlation was strongest when using the SSIOSS tool, with a converted correlation coefficient of *r* = 0.35 (95% CI, 0.31, 0.39), but relatively smaller when using the BSI-CV tool (*r* = 0.24, 95% CI: 0.16, 0.32) or other measurement tools (*r* = 0.26, 95% CI: 0.22, 0.30) (Figure 4).

### 3.5. Sensitivity analysis

**Figure 5:**
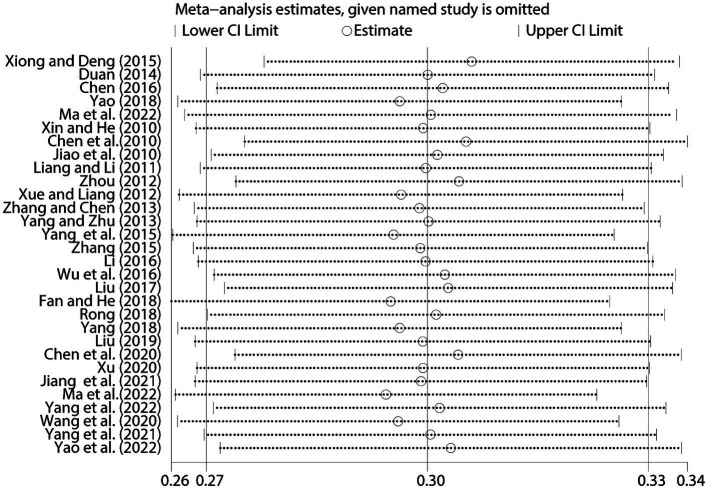
Sensitivity analysis between NLE and SI.

A sensitivity analysis was performed to evaluate the robustness of our study. This analysis involved systematically removing one individual study at a time and recalculating the summary correlation coefficient. The results of the sensitivity analysis revealed minimal variation in the overall correlation coefficient between NLE and SI, indicating the stability of our findings (Figure 5).

### 3.6. Publication bias

**Figure 6:**
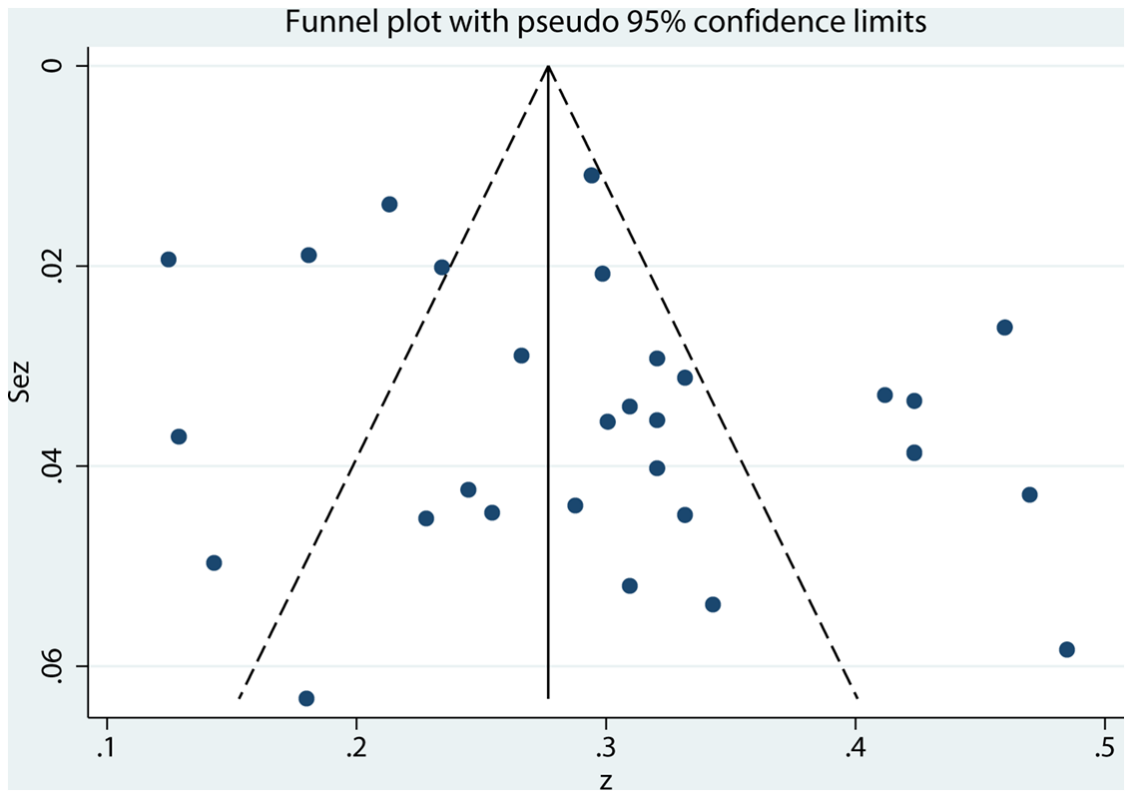
Funnel plot between NLE and SI.

**Figure 7:**
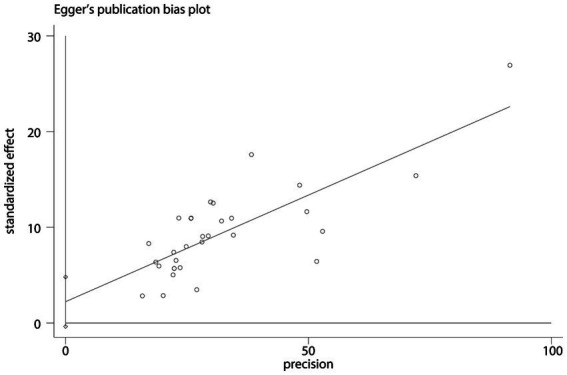
Egger plot between NLE and SI.

Subjective assessment of funnel plot symmetry for the summary correlation coefficient between NLE and SI (Figure 6) was challenging to determine. Egger’s regression test showed no statistically significant asymmetry (*t* = 1.77, *p* = 0.088) (Figure 7), indicating no significant evidence of publication bias detected.

## 4. Discussion

In recent years, adolescent SI remains a significant public health issue globally (15). NLE has been recognized as a significant factor in problem behaviors and has received increased attention (29). However, the findings from numerous studies on the association between NLE and SI remain inconclusive (30, 31, 71, 72). The reason for this controversy may be due to the different impacts of the types of NLE that adolescents have experienced in the past on SI. However, the findings of this meta-analysis revealed a significant and moderate correlation between NLE and SI among Chinese adolescents. The reasons for this analysis are as follows: firstly, according to the stress-vulnerability model proposed by Mann et al. (73), stress factors and quality factors jointly influence an individual’s suicidal ideation. NLE as an important component of stress factors can cause stress in individuals after cognitive evaluation. When the stress intensity exceeds the individual’s adaptive and coping abilities and cannot effectively solve the problem, it leads to an increase in adolescents’ stress levels. The failure of stress response to events is considered by Miller et al. (7) as a possible mechanism for adolescents to generate SI. Secondly, adolescence is a sensitive period for the physiological and psychological development of adolescents, during which significant neurological and biological changes occur (74–76). NLE as stress factors may reshape the structure of the cerebral cortex, amygdala, prefrontal cortex, and hippocampus, altering an individual’s neuro-psychological state, such as causing sustained sensitization of the hypothalamic-pituitary-adrenal axis (HPA) (77), increasing the fragility of the harmful effects of stress, and this change is long-term and thorough, extending to adolescence, adulthood, and even old age (78). Some studies have suggested that the high reactivity of the HPA axis is strongly associated with SI (79, 80). At the same time, adolescents who have experienced adverse life events during adolescence have higher levels of 3-methoxy-4-hydroxyphenylglycole (MHPG) in their cerebrospinal fluid (CSF-MHPG) and urine levels of noradrenaline/adrenaline (NA/A), and lower levels of cortisol (77), thus leading to HPA axis dysfunction and increased potential risk of adolescent SI (81). Finally, as Chinese society progresses, teenagers face more intricate social connections, higher educational expectations, and additional social and family stressors. Consequently, this leads to heightened levels of NLE and subsequent psychological issues like anxiety, depression, and social isolation (82). Prolonged exposure to these adverse feelings could potentially heighten the likelihood of SI (83).

### 4.1. The moderating effect of region

The findings of this study unveiled a significant moderating effect of regional disparities on the association between NLE and SI. Notably, a robust correlation between NLE and SI was observed among adolescents residing in Western China, while comparatively moderate correlations were noted in the Central and Eastern regions. This disparity may be attributed to the economic underdevelopment of the Western region, where parents often work away from home, resulting in limited emotional communication within families and rendering adolescents more susceptible to NLE, such as interpersonal and familial conflicts (84). Adverse family and interpersonal relationships, in turn, augment the risk of suicidal ideation among adolescents (85, 86). Therefore, the correlation between NLE and SI appears to be more pronounced among adolescents in Western China. This also highlights the need for greater efforts in strengthening psychological health interventions for adolescents in Western regions of China.

### 4.2. The moderating effect of age

This study found that age did not moderate the relationship between NLE and SI among Chinese adolescents. On one hand, this may be attributed to the fact that university students and high school students are exposed to similar cultural and social environments, making the impact of external factors relatively consistent. On the other hand, university students and high school students are relatively similar in terms of age and psychological development (87). During this period, they are separated from their parents and must face the challenges of studying and living independently, dealing with complex interpersonal relationships, and heavy academic pressures. These NLE further increase the risk of SI. This also serves as a reminder that as the main gathering place for adolescents, schools should strive to create a harmonious and favorable environment, instill correct academic and learning attitudes in students, reduce their academic burden, and minimize the occurrence of adverse life events.

### 4.3. The moderating effect of publication year

There was no notable moderating impact of the year of publication on the relationship between NLE and SI in Chinese teenagers. This suggests that the correlation between NLE and SI seems to remain consistent over time within this group. Nevertheless, it is important to acknowledge that restricting the analysis to studies published in the last 13 years could potentially restrict the applicability of the results, as they may not fully capture the long-term trends or changes in this field.

### 4.4. The moderating effect of SI measurement tools

The measurement of SI moderated the correlation between NLE and SI. When using BSI-CV and other (*r* = 0.24, 0.26) to measure SI, the positive correlation was smaller compared to using SSIOSS measurement (*r* = 0.35). One potential explanation for this finding could be that the BSI-CV and other measurement tools used in the included studies may not have been fully adapted to the Chinese mainland context, resulting in relatively small effect sizes. This could be due to differences in cultural norms, language, or other contextual factors that may impact the measurement and interpretation of NLE and SI in Chinese adolescents. Furthermore, as this study classified SI measurement tools other than SSIOSS and BSI-CV as other measurements, it is necessary to conduct additional investigation to ascertain if the association between NLE and SI is influenced by other infrequently utilized SI measurement tools.

### 4.5. Advantages and limitations

The purpose of this review was to gather and thoroughly examine existing data to clarify the connection between NLE and SI in Chinese teenagers. Most of the studies included in the analysis had strong sample sizes and a significant number of participants, which greatly enhanced the credibility of the research results. The insights gleaned from this research may contribute novel perspectives to the field of SI prevention and clinical interventions for Chinese adolescents, thereby informing evidence-based practices and interventions in this context.

Nevertheless, it is crucial to recognize the constraints of this research. All the literature included in this analysis primarily comprised cross-sectional observational studies. Hence, our discoveries merely imply a connection between NLE and SI, without being able to establish a cause-and-effect relationship. Furthermore, the scope of this study was limited to Chinese adolescents, neglecting the investigation of other age categories, which consequently restricts the applicability of the findings. Thirdly, it should be underscored that we refrained from conducting an in-depth analysis of the specific typology of NLE encountered by adolescents, a factor that may potentially impact the comprehensiveness of the outcomes obtained. In conclusion, this research solely examined the straightforward association between NLE and SI.

## 5. Conclusion

The results of this extensive systematic review and meta-analysis clearly emphasize a strong and statistically significant correlation between NLE and SI among Chinese adolescents. It is especially important to give particular focus to teenagers in the Western areas of China, as they might face a greater occurrence of NLE, consequently raising their vulnerability to the danger of SI. Thus, early attention to NLE in adolescents and targeted interventions can directly reduce the occurrence of SI. Adolescence is a distinct period during which young individuals possess distinctive mental and physical traits and will encounter various NLE in their lives. To reduce the incidence of NLE, it is important for schools, families, and society to collaborate and establish a conducive and amicable atmosphere. Simultaneously, it is crucial to enhance primary healthcare and establish appropriate psychological counseling centers to offer prompt support for the mental well-being of teenagers when confronted with stressful life occurrences or noteworthy situations like troubled social connections or strained parent-child relationships, thereby diminishing the likelihood of SI.

## Data availability statement

The original contributions presented in the study are included in the article/Supplementary material, further inquiries can be directed to the corresponding author.

## Author contributions

PY and BY participated in the study selection and evaluation of quality. The data conceptualization, literature search, critical appraisal, and statistical analysis were carried out by XBH and QYY. BY reviewed and edited the manuscript. All authors contributed to the article and approved the submitted version.

## Conflict of interest

The authors declare that the research was conducted in the absence of any commercial or financial relationships that could be construed as a potential conflict of interest.

## Publisher’s note

All claims expressed in this article are solely those of the authors and do not necessarily represent those of their affiliated organizations, or those of the publisher, the editors and the reviewers. Any product that may be evaluated in this article, or claim that may be made by its manufacturer, is not guaranteed or endorsed by the publisher.
