# Supplementary material for: Correlations between negative life events and suicidal ideation among Chinese adolescents: a meta-analysis
Source: Front Psychiatry. 2023 Sep 14;14:1201786. doi: 10.3389/fpsyt.2023.1201786 (PMC10539609; doi:10.3389/fpsyt.2023.1201786)
Supplement: Supplementary file 1 [file Data_Sheet_1.zip › Supplementary_Material/Additional file.docx]

**Additional file**

**Supplementary material 1:** Search strategy used in the current systematic review and meta-analysis

**Supplementary material 2:** Methodological quality assessment of the literature

***PUBMED***

#1

“suicidal ideation”[MeSH Terms] OR "suicide, attempted"[MeSH Terms] OR "self injurious behavior"[MeSH Terms] OR "self injurious behavior"[MeSH Terms] OR "self injurious behavior"[MeSH Terms]

#2

"self-harm"[Title/Abstract] OR "self harm*"[Title/Abstract] OR "self injur*"[Title/Abstract] OR "suicid*"[Title/Abstract] OR "para suicid*"[Title/Abstract] OR "parasuicid*"[Title/Abstract] OR "suicidal behav*"[Title/Abstract] OR "suicide attempt"[Title/Abstract] OR "self injur*"[Title/Abstract] OR "self inflict*"[Title/Abstract] OR "suicidal ideation"[Title/Abstract]

#3

"Life Change Events"[MeSH Terms]

#4

"life experience"[Title/Abstract] OR "life events"[Title/Abstract] OR "stressful life events"[Title/Abstract] OR "negtive life events"[Title/Abstract]

#5

"adolescent"[MeSH Terms]

#6

"Young Person"[Title/Abstract] OR "Young People"[Title/Abstract] OR "Young Adult"[Title/Abstract] OR "teen*"[Title/Abstract] OR "adolesc*"[Title/Abstract] OR "youth*"[Title/Abstract] OR "student*"[Title/Abstract] OR "middle school student"[Title/Abstract] OR "College student"[Title/Abstract] OR "university student"[Title/Abstract] OR "freshman"[Title/Abstract] OR "sophomore"[Title/Abstract] OR "junior student"[Title/Abstract] OR "senior student"[Title/Abstract] OR "undergraduate"[Title/Abstract]

#7

(RCT or “random controlled trial” [Title/Abstract])

#8

(#1 OR #2) AND (#3 OR #4) AND (#5 OR #6) NOT #7

***Web of Sci***

#1

TS= (“attempted suicide” OR “deliberate self harm” OR “self injurious behavior” OR “self-injury” OR “self-harm” OR “self harm*” OR “self-injur*” OR “self injur*” OR “self-cut*” OR “self-destruct” OR “nonsuicidal self-injur*” OR “non-suicidal self injur*” OR “deliberate self harm” OR “self-mutil*” OR overdos* OR “self-inflicted injur*” OR “self inflicted injur*” OR suicid* OR “para-suicid*” OR parasuicid* OR “suicidal behav*” OR Suicide OR “suicide attempt” OR “self?harm*” OR “self?injur*” OR “self?poison*” OR “self?inflict*” OR “Suicidal Ideation”)

#2

**TS=** (“life experience” OR “life events” OR “stressful life events” OR “negtive life events” OR “life change events”)

#3

**TS=** (“Young Person” OR “Young People” OR “Young Adult” OR teen* OR adolesc* OR youth* OR student* OR “middle school student” OR “College student” OR “university student” OR freshman OR sophomore OR “junior student” OR “senior student” OR undergraduate)

#4

#1 AND #2 AND #3 **AND LANGUAGE: (English)**

***EMBASE***

#1

'attempted suicide'/exp OR 'deliberate self harm'/exp OR 'self injurious behavior'/exp OR 'self-injury'/exp OR 'suicidal ideation'/exp

#2

'attempted suicide':ab,ti OR 'self injurious behavior':ab,ti OR 'self-injury':ab,ti OR 'self-harm':ab,ti OR 'self harm*':ab,ti OR 'self-injur*':ab,ti OR 'self injur*':ab,ti OR 'self-cut*':ab,ti OR 'self-destruct':ab,ti OR 'nonsuicidal self-injur*':ab,ti OR 'non-suicidal self injur*':ab,ti OR 'deliberate self harm':ab,ti OR 'self-mutil*':ab,ti OR overdos*:ab,ti OR 'self-inflicted injur*':ab,ti OR 'self inflicted injur*':ab,ti OR suicid*:ab,ti OR 'para-suicid*':ab,ti OR parasuicid*:ab,ti OR 'suicidal behav*':ab,ti OR suicide:ab,ti OR 'suicide attempt':ab,ti OR 'self?harm*':ab,ti OR 'self?injur*':ab,ti OR 'self?poison*':ab,ti OR 'self?inflict*':ab,ti OR 'suicidal ideation':ab,ti

#3

'life change events'/exp

#4

'life experience':ab,ti OR 'life events':ab,ti OR 'stressful life events':ab,ti OR 'negtive life events':ab,ti OR 'life change events':ab,ti

#5

adolescent/exp

#6

'Young Person':ab,ti OR 'Young People':ab,ti OR 'Young Adult':ab,ti OR 'teen*':ab,ti OR 'adolesc*':ab,ti OR 'youth*':ab,ti OR 'student*':ab,ti OR 'middle school student':ab,ti OR 'College student':ab,ti OR 'university student':ab,ti OR 'freshman':ab,ti OR sophomore:ab,ti OR 'junior student':ab,ti OR undergraduate:ab,ti OR 'senior student':ab,ti

#7

(#1 OR #2) AND (#3 OR #4) AND (#5 OR #6)

#8

limit to (human and (article or article in press)

**Supplementary material 2:** **Methodological quality assessment of the literature**

S2： Agency for Health Care Research and Quality (AHRQ)

| Study | Year | ① | ② | ③ | ④ | ⑤ | ⑥ | ⑦ | ⑧ | ⑨ | ⑩ | ⑪ |
| --- | --- | --- | --- | --- | --- | --- | --- | --- | --- | --- | --- | --- |
| Xiong and Deng | 2015 | Y | Y | Y | Y | U | N | Y | N | N | Y | N |
| Duan | 2014 | Y | Y | Y | Y | U | N | N | N | N | Y | N |
| Chen | 2016 | Y | Y | Y | Y | U | Y | Y | Y | N | N | N |
| Yao | 2018 | Y | Y | Y | Y | U | Y | N | N | N | Y | N |
| Ma et al. | 2022 | Y | Y | Y | Y | U | Y | Y | Y | N | Y | N |
| Xin and He | 2010 | Y | Y | Y | Y | U | Y | N | Y | N | Y | N |
| Xin and He | 2010 | Y | Y | Y | Y | U | N | N | N | N | Y | N |
| Jiao et al | 2010 | Y | Y | Y | Y | U | Y | Y | Y | N | Y | N |
| Liang and Li | 2011 | Y | Y | Y | Y | U | Y | N | N | N | N | N |
| Zhou | 2012 | Y | Y | Y | Y | U | Y | Y | N | N | Y | N |
| Xue and Liang | 2012 | Y | Y | Y | Y | U | N | N | N | N | Y | N |
| Xue and Liang | 2013 | Y | Y | Y | Y | U | N | Y | N | N | N | N |
| Yang and Zhu | 2013 | Y | Y | Y | Y | U | Y | N | Y | N | N | N |
| Yang et al | 2015 | Y | Y | Y | Y | U | Y | N | N | N | N | N |
| Zhang | 2015 | Y | Y | Y | Y | U | Y | Y | Y | Y | Y | N |
| Li | 2016 | Y | Y | Y | Y | U | N | N | N | N | Y | N |
| Wu et al | 2016 | Y | Y | Y | Y | U | Y | Y | Y | Y | Y | N |
| Liu | 2017 | Y | Y | Y | Y | U | Y | N | N | N | N | N |
| Fan and He | 2018 | Y | Y | Y | Y | U | Y | N | Y | Y | N | N |
| Rong | 2018 | Y | Y | Y | Y | U | N | N | N | N | Y | N |
| Lu et al | 2020 | Y | Y | Y | Y | U | N | Y | N | N | Y | N |
| Yang | 2018 | Y | Y | Y | Y | U | Y | Y | N | N | N | N |
| Liu | 2019 | Y | Y | Y | Y | U | Y | Y | Y | N | Y | N |
| Chen et al | 2020 | Y | Y | Y | Y | U | Y | Y | Y | N | Y | N |
| Xu | 2020 | Y | Y | Y | Y | U | N | N | Y | N | N | N |
| Jiang et al | 2021 | Y | Y | Y | Y | U | N | N | Y | N | Y | N |
| Ma et al | 2022 | Y | Y | Y | Y | U | Y | Y | Y | N | N | N |
| Yang et al | 2022 | Y | Y | Y | Y | U | Y | N | Y | N | N | N |
| Wang et al | 2020 | Y | Y | Y | Y | U | Y | N | Y | Y | N | N |
| Yang et al | 2021 | Y | Y | Y | Y | U | N | N | Y | N | N | N |
| Yao et al | 2022 | Y | Y | Y | Y | U | Y | Y | Y | N | Y | N |

Y: Yes; N:No; U: Uclear; ①Define the source of information (survey, record review); ②List inclusion and exclusion criteria for exposed and unexposed subjects(cases and controls) or refer to previous publications; ③Indicate time period used for identifying patients; ④indicate whether or not subjects were consecutive if not population-based; ⑤indicate if evaluators of subjective components of study were masked to other aspects of the status of the participants; ⑥Describe any assessments undertaken for quality assurance purposes(e.g., test/retest of primary outcome measurements); ⑦Explain any patient exclusions from analysis; ⑧Describe how confounding was assessed and/or controlled; ⑨If applicable, explain how missing data were handled in the analysis; ⑩Summarize patient response rates and completeness of data collection; ⑪Clarify what follow-up, was expected and percentage of patients for which incomplete data or follow-up was obtained.
